# Benchmarking large language model-based agent systems for clinical decision tasks

**DOI:** 10.1038/s41746-026-02443-6

**Published:** 2026-02-18

**Authors:** Yunsong Liu, Zunamys I. Carrero, Xiaofeng Jiang, Dyke Ferber, Georg Wölflein, Li Zhang, Sanddhya Jayabalan, Tim Lenz, Zhouguang Hui, Jakob Nikolas Kather

**Affiliations:** 1 Department of Radiation Oncology, National Cancer Center/National Clinical Research Center for Cancer/Cancer Hospital, Chinese Academy of Medical Sciences and Peking Union Medical College, Beijing, China; 2 Else Kroener Fresenius Center for Digital Health, Faculty of Medicine and University Hospital Carl Gustav Carus, TUD Dresden University of Technology, Dresden, Germany; 3 Department of Thoracic Surgery, Sichuan Clinical Research Center for Cancer, Sichuan Cancer Hospital & Institute, Sichuan Cancer Center, University of Electronic Science and Technology of China (UESTC), Chengdu, China; 4 Medical Oncology, National Center for Tumor Diseases (NCT), University Hospital Heidelberg, Heidelberg, Germany; 5 School of Computer Science, University of St Andrews, St Andrews, UK; 6 Department of VIP Medical Services, National Cancer Center/National Clinical Research Center for Cancer/Cancer Hospital, Chinese Academy of Medical Sciences and Peking Union Medical College, Beijing, China; 7 Department of Medicine I, Faculty of Medicine and University Hospital Carl Gustav Carus, TUD Dresden University of Technology, Dresden, Germany; 8 Pathology & Data Analytics, Leeds Institute of Medical Research at St James’s University of Leeds, Leeds, UK

**Keywords:** Business and industry, Computational biology and bioinformatics, Health care, Mathematics and computing, Medical research

## Abstract

Agentic artificial intelligence (AI) systems, designed to autonomously reason, plan, and invoke tools, have shown promise in healthcare, yet systematic benchmarking of their real-world performance remains limited. In this study, we evaluate two such systems: the open-source OpenManus, built on Meta’s Llama-4 and extended with medically customized agents; and Manus, a proprietary agent system employing a multistep planner-executor-verifier architecture. Both systems were assessed across three benchmark families: *AgentClinic*, a stepwise dialog-based diagnostic simulation; *MedAgentsBench*, a knowledge-intensive medical QA dataset; and *Humanity’s Last Exam* (HLE), a suite of challenging text-only and multimodal questions. Despite access to advanced tools (e.g., web browsing, code development and execution, and text file editing) agent systems yielded only modest accuracy gains over baseline LLMs, reaching 60.3% and 28.0% in AgentClinic MedQA and MIMIC, 30.3% on MedAgentsBench, and 8.6% on HLE text. Multimodal accuracy remained low (15.5% on multimodal HLE, 29.2% on AgentClinic NEJM), while resource demands increased substantially, with >10× token usage and >2× latency. Although 89.9% of hallucinations were filtered by in-agent safeguards, hallucinations remained prevalent. These findings reveal that current agentic designs offer modest performance benefits at significant computational and workflow cost, underscoring the need for more accurate, efficient, and clinically viable agent systems.

## Introduction

Clinical decision-making entails complex, data-intensive, and often uncertain judgments, resulting in excessive workload and exceeding the cognitive limits of many clinicians. For more than two decades, the medical AI community has pursued computational support for clinical decision-making. Initial efforts used machine learning algorithms in clinical decision support system (CDSS), to learn directly from large-scale electronic health records, imaging, and omics data to perform disease diagnosis, risk stratification, and treatment recommendation^1,2^. Subsequently, deep learning models revolutionized tasks such as medical image interpretation and sequential data analysis by capturing complex, high-dimensional features^1,2^. Advances in natural language processing (NLP) further enabled automatic extraction of meaningful insights from unstructured clinical text, bridging structured databases with clinicians’ narrative workflows^1,2^. Yet core challenges persisted, such as narrow tasks, domain shift, limited generalizability and fragile NLP schemas. Critically, most tools supported isolated actions rather than multi-step clinical reasoning, constraining real-world clinical integration.

Recently, large language models (LLMs) have introduced powerful general-purpose reasoning and problem-solving capabilities, showing promise in numerous clinical tasks such as clinical text summarization, medical question answering, research support and medical education^3–5^, marking a new era for AI-driven clinical decision support. For instance, zero-shot GPT-4 achieved 86.1% accuracy on the United States Medical Licensing Examination (USMLE) without domain-specific fine-tuning^6^. Through medical-domain fine-tuning, smaller models such as Med-PaLM 2 reached comparable performance (86.5%)^7^. However, healthcare delivery in the real world presents complex challenges, including multi-round patient–doctor interactions, diagnostic reasoning, treatment planning, and managing rare or ambiguous clinical presentations. A meta-analysis of 83 diagnostic studies found that generative AI systems averaged only 52% accuracy, substantially lower than expert clinicians and insufficient for reliable clinical deployment^8^. Within a virtual electronic health record environment, the best-performing model among 12 state-of-the-art LLMs achieved a task success rate of 69.7%^9^. In simulated dialog-based diagnostic scenarios reflective of real-world complexity, the diagnostic accuracy decreased significantly compared to vignettes and even advanced models such as GPT-4o achieved only 34.2% accuracy^10,11^. Furthermore, on challenging medical questions, zero-shot GPT-4o dropped to 18.0% accuracy^12^. These findings underscore a significant gap between current zero-shot LLM capabilities and the demands of real-world clinical problem-solving, posing potential risks in clinical settings.

To address these limitations, the field is moving beyond single-pass prompting of LLMs toward agentic architectures. AI agents are autonomous entities engineered for goal-directed task execution within bounded digital environments, capable of utilizing external tools, applying sequential reasoning, and integrating real-time information^13^. In contrast, agentic AI systems represent intelligent architectures where multiple specialized agents collaborate through structured communication, shared memory, and dynamic role assignment to achieve complex, high-level objectives^13^. In contemporary implementations, LLMs serve as the core reasoning and perception engines, enabling these systems to plan, act, and adapt across tasks^13,14^. Such agent systems outperform or accomplish tasks beyond the reach of single LLM approaches in multiple areas such as programming^15^, scientific idea generation^16,17^, and disease prediction^18^. Recently, generalist agent systems such as Manus and its open-source counterpart, OpenManus, have gained prominence^19^. These systems feature comprehensive agent architectures with multi-tool and multi-step designs. Manus reportedly surpasses GPT-4 by up to 12.2% in accuracy on the GAIA benchmark^20^. These capabilities suggest that clinical agents could systematically collect additional data, reason effectively, and develop structured action plans, potentially improving clinical performance^21^. However, systematic evaluation of these agentic AI systems, specifically in healthcare contexts, remains sparse.

In this study, we provide a systematic evaluation of LLM-based agent systems on clinical tasks. Specifically, we assess the performance of Manus and OpenManus (both baseline and medically tailored versions) against state-of-the-art zero-shot LLMs, including GPT-4.1, Qwen-3, Gemma-3, MedGemma, and Llama-4. The evaluation spans three distinct healthcare-focused benchmarks: AgentClinic (simulated stepwise doctor-patient dialogs), MedAgentsBench (knowledge-intensive question answering), and Humanity’s Last Exam (HLE) (textual and multimodal medical questions). Metrics evaluated include accuracy, computational efficiency, workflow complexity, and hallucination mitigation, aiming to quantify the incremental value and associated costs of deploying generalist agent systems within healthcare. This comprehensive benchmarking aims to inform future research directions, guiding effective and safe implementation of agentic AI systems in clinical practice.

## Results

### Agent systems yield marginal improvement over baseline LLMs

**Fig. 1:**
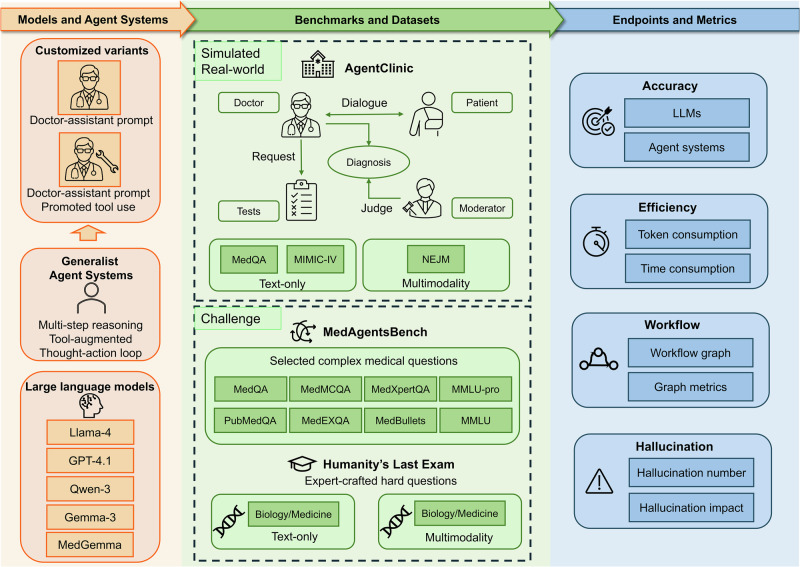
Workflow graph depicting the overall process of the present study. Baseline LLMs (Llama-4, Qwen-3, Gemma-3, GPT-4.1, and MedGemma) and generalist agent systems, with customized variants, were evaluated across text-only and multimodal tasks. Three benchmarks were used: AgentClinic (diagnostic tasks with simulated dialogs from MedQA, MIMIC-IV, and NEJM case challenges), MedAgentsBench (a knowledge-intensive medical question set), and Humanity’s Last Exam (a suite of challenging text-only and multimodal questions). Primary and secondary endpoints included accuracy, efficiency (token/time), workflow complexity (workflow graph and complexity), and safety (hallucination frequency and impact).

The overall workflow of the present study is illustrated in Fig. 1. We first evaluated the accuracy of agent systems across multiple benchmarks, encompassing LLM-simulated dialog-based diagnostic tasks (AgentClinic, MedQA, and MIMIC-IV datasets), challenging medical questions (MedAgentsBench, HLE), and multimodal medical tasks (AgentClinic NEJM dataset and HLE multimodal items).

**Fig. 2:**
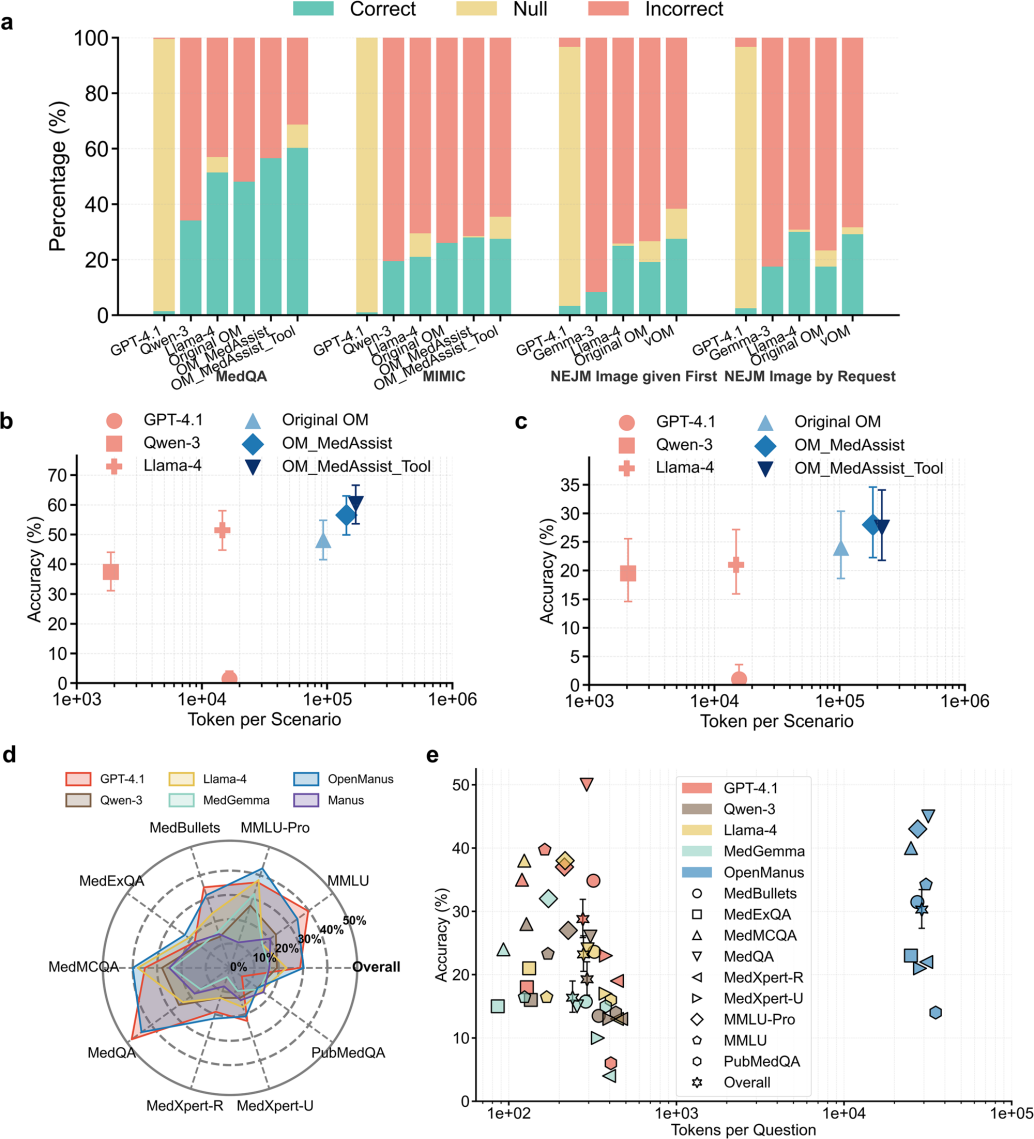
Performance of LLMs and agent systems on AgentClinic and MedAgentsBench. **a** Distribution of diagnostic outcomes (correct, incorrect, and null) for LLMs and the four agent systems in AgentClinic. The final answer was judged after a maximum of 20 dialogs; “null” indicates that the model did not provide a diagnosis after reaching this limit. Notably, MedGemma is not suitable for AgentClinic, as it failed to produce an answer in all cases. Four agent systems – original OpenManus (Original OM), OpenManus with a physician-assistant prompt (OM_MedAssist), further encouraged to use tools (OM_MedAssist_Tool), and equipped with a medical-vision tool (vOM) – were evaluated. **b** Accuracy versus average tokens per scenario on AgentClinic-MedQA, showing the trade-off between reasoning cost and performance for each model. OM_MedAssist_Tool achieved the highest accuracy (60.3%) but required substantially high tokens. **c** Accuracy versus average tokens per scenario on AgentClinic–MIMIC, illustrating similarly low overall accuracy. OM_MedAssist achieved the highest accuracy of 28.0%, again with high token consumption. **d** Radar plot of model accuracies on MedAgentsBench across each individual dataset and the pooled overall accuracy, calculated as total correct divided by total cases. Two agent systems—OpenManus and Manus—were evaluated. OpenManus achieved the highest overall accuracy (30.3%), followed by GPT-4.1 (28.8%). **e** Accuracy versus average tokens per question across each MedAgentsBench dataset and the overall pooled set. Despite only minimal accuracy advantages, OpenManus incurred extremely high token usage—approximately 100 times more than its backbone Llama-4. Error bars indicate 95% confidence intervals. OM OpenManus.

To determine an efficient reasoning depth for OpenManus in dialog-based diagnostics, we systematically varied the maximum number of reasoning steps (from 1 to 20) using 20 pilot cases from the MedQA dataset (Fig. S1a). Accuracy peaked at five and seven steps; the five-step configuration was selected for subsequent AgentClinic experiments to balance computational efficiency and performance. Under this configuration, baseline LLMs achieved accuracies of 1.4% (GPT-4.1), 37.4% (Qwen-3), and 51.4% (Llama-4) on the complete MedQA subset (Fig. 2a, b and Table 1). MedGemma failed to produce an answer for all AgentClinic tasks, reaching the step limit in every scenario. While the original OpenManus (48.1%) did not surpass its backbone LLM (Llama-4), performance improved with a physician-assistant prompt (OpenManus_MedAssist, 56.5%) and further increased with explicit tool integration (OpenManus_MedAssist_Tool, 60.3%). However, these improvements did not reach statistical significance compared with Llama-4 (Fig. S2b). A similar pattern was observed in the MIMIC-IV dataset, where the baseline LLMs achieved accuracies of 1.0% (GPT-4.1), 19.5% (Qwen-3), and 24.0% (Llama-4), whereas the original OpenManus reached 24.0%, and enhancements with OpenManus_MedAssist and OpenManus_MedAssist_Tool increased accuracy to 28.0% and 27.5%, respectively, without statistical significance (Fig. 2a, c; Table 1 and Fig. S1c).

**Table 1:** Performance and efficiency in text-based tasks

Model	AgentClinic-MedQA			AgentClinic-MIMIC			MedAgentsBench			Humanity’s last exam		
	Accuracy (%)	Tokens	Time (s)	Accuracy (%)	Tokens	Time (s)	Accuracy (%)	Tokens	Time (s)	Accuracy (%)	Tokens	Time (s)
GPT-4.1	1.4 (0.5–4.0)	16,553	NA	1.0 (0.3–3.6)	15,712	NA	28.8 (25.8–31.9)	274	NA	5.9 (3.5–9.8)	1226	NA
Qwen-3	37.4 (31.2–44.0)	1873	NA	19.5 (14.6–25.5)	2046	NA	19.3 (16.8–22.2)	291	NA	7.7 (4.8–11.9)	5396	NA
MedGemma	NA	NA	NA	NA	NA	NA	16.4 (14.4–19.0)	240	NA	8.1 (5.2–12.5)	1815	NA
Llama-4	51.4 (44.7–58.1)	14,576	32.2	21.0 (15.9–27.2)	14,885	25.0	23.2 (20.5–26.1)	276	0.08	5.0 (2.8–8.7)	1061	7.0
Original OpenManus	48.1 (41.5–54.8)	92,954	39.0	24.0 (18.6–30.4)	102,604	41.9	30.3 (27.3–33.4)	29,150	20.0	8.6 (5.5–13.0)	14,837	11.3
OpenManus_MedAssist	56.5 (49.8–63.0)	142,316	54.6	28.0 (22.2–34.6)	184,227	102.2	NA	NA	NA	NA	NA	NA
OpenManus_MedAssist_Tool	60.3 (53.6–66.6)	168,957	68.9	27.5 (21.8–34.1)	217,425	92.5	NA	NA	A	NA	NA	NA
Manus	NA	NA	NA	NA	NA	NA	16.1 (13.8–18.7)	NA	NA	7.7 (4.8–11.9)	NA	NA

Tokens refer to the average number of tokens consumed per scenario. Time refers to the average response time per scenario. Four agent systems were evaluated: the original OpenManus, OpenManus with a physician-assistant prompt (OpenManus_MedAssist), OpenManus further encouraged to use tools (OpenManus_MedAssist_Tool), and Manus. OpenManus_MedAssist and OpenManus_MedAssist_Tool are designed for interactive diagnostic tasks and are therefore only applicable to AgentClinic, whereas Manus, as a commercial closed-source system, cannot participate in interactive AgentClinic workflows. MedGemma failed to produce answers on the AgentClinic benchmark. Time measurements are available only for comparisons between OpenManus-based agent systems and their backbone model, Llama-4. Values in brackets denote 95% confidence intervals. NA indicates non-applicable data.

Turning to knowledge-heavy multiple-choice tasks, we evaluated agent reasoning depth using 20 items from MedExQA, finding best accuracy at ten reasoning steps (Fig. S1b). On the complete 862-item MedAgentsBench HARD set, OpenManus achieved 30.3% accuracy, marginally exceeding GPT-4.1 (28.8%) and surpassing it in six of the nine individual datasets. Comparatively, Llama-4, MedGemma and Manus showed lower accuracies of 23.2, 16.4 and 16.1%, respectively (Fig. 2d, e and Table 1). OpenManus achieved a statistically significant improvement over its backbone Llama-4 (*p* < 0.001) but not for GPT-4.1 (*p* = 0.871) (Fig. S1d). Across these datasets, answer-choice distributions were generally balanced, and balanced accuracy closely matched overall accuracy (Fig. S3a, b).

**Fig. 3:**
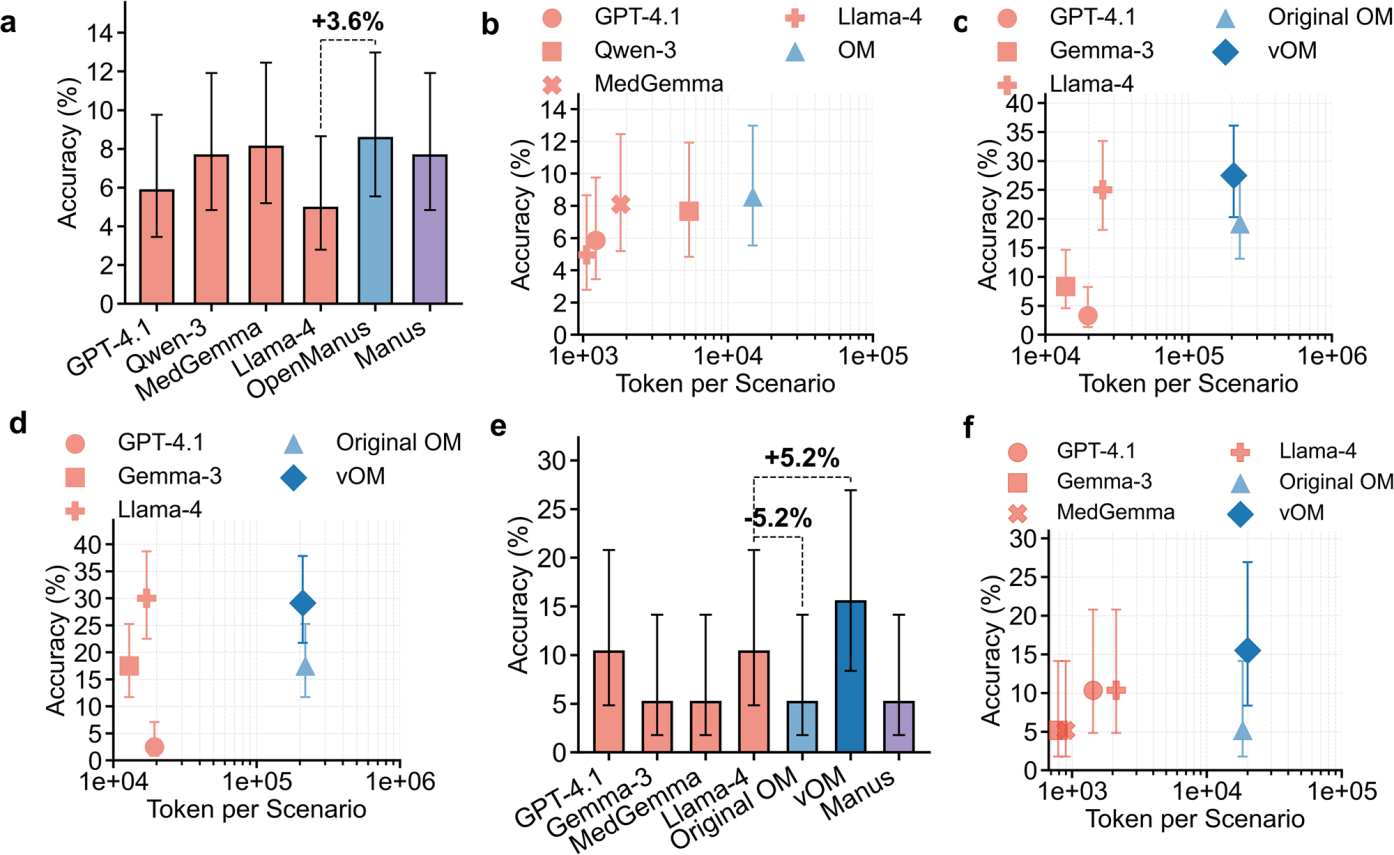
Performance of LLMs and agent systems on humanity’s last exam and multimodality benchmarks. **a** Accuracy on Humanity’s last exam (HLE) text-only tasks. Two agent systems—OpenManus and Manus—were evaluated. Agent systems achieved accuracy comparable to standalone LLMs, with OpenManus improving upon its backbone Llama-4 model by 3.6%. **b** Accuracy versus average tokens per scenario on HLE, illustrating the trade-off between reasoning cost and performance across models. **c** Accuracy versus average token usage per scenario on the AgentClinic-NEJM multimodal dataset when images were provided at the beginning of each case. Two agent systems were evaluated: the original OpenManus and vOpenManus (OpenManus equipped with a medical-vision tool). vOpenManus achieved slightly higher accuracy (27.5%) compared with its backbone Llama-4 (25%), but with substantially higher token usage. **d** Scatter plot of accuracy versus token usage on the AgentClinic-NEJM multimodal dataset when images were supplied only upon the model’s request. Original OpenManus and vOpenManus were evaluated. Llama-4 achieved the highest accuracy (30%), outperforming both agent systems. **e** Accuracy on HLE multimodal tasks. Three agent systems – original OpenManus, vOpenManus and Manus—were evaluated. vOpenManus achieved the highest accuracy, improving upon its backbone Llama-4 by 5.2%. **f** Accuracy versus average token usage per scenario on HLE multimodal tasks. While vOpenManus provided performance gains, the improvement came at the cost of substantially increased token consumption. Error bars indicate 95% confidence intervals. OM OpenManus.

In the Biology/Medicine subset of HLE, designed to minimize the effect of shortcut reasoning, agent-based approaches provided limited gains in accuracy: results clustered at 5.0% (Llama-4), 5.9% (GPT-4.1), 7.7% (Qwen-3 and Manus), 8.1% (MedGemma) and 8.6% (OpenManus) (Fig. 3a, b and Table 1), using the same step configuration identified previously. No statistically significant differences were observed among model performances (*p* = 0.589) (Fig. S2a).

We further evaluated multimodal performance in AgentClinic using two senarios for image input: (1) images presented at the scenario start and (2) images provided upon request from the doctor agent. In the first scenario, vOpenManus achieved the highest accuracy (27.5%), followed by Llama-4 (25.0%), original OpenManus (19.2%), Gemma-3 (8.3%), and GPT-4.1 (3.3%) (Fig. 3c and Table 2). GPT-4.1 exhibited a high null-answer rate of 93.3%, whereas Gemma-3, Llama-4, and original OpenManus left fewer than 10% of cases unanswered, and vOpenManus had a 10.8% null-answer rate (Fig. 2a). In the second scenario, Llama-4 led with 30.0% accuracy, followed closely by vOpenManus (29.2%), original OpenManus (17.5%), Gemma-3 (17.5%), and GPT-4.1 (2.5%) (Fig. 3d and Table 2). All models except GPT-4.1 (94.1% null-answer rate) produced null answers in fewer than 10% of cases (Fig. 2a). vOpenManus did not achieve statistically significant improvement over its backbone Llama-4 in either scenario (Fig. S2e, f).

**Table 2:** Performance and efficiency in multimodal tasks

Model	AgentClinic NEJM Image given first			AgentClinic NEJM Image by request			Humanity’s last exam		
	Accuracy (%)	Tokens	Time (s)	Accuracy (%)	Tokens	Time (s)	Accuracy (%)	Tokens	Time (s)
GPT-4.1	3.3 (1.3–8.3)	19,783	NA	2.5 (0.8–7.1)	19,320	NA	10.3 (4.8–20.8)	1433	NA
Gemma-3	8.3 (4.6–14.7)	13,852	NA	17.5 (11.7–25.3)	12,866	NA	5.2 (1.8–14.1)	790	NA
MedGemma	NA	NA	NA	NA	NA	NA	5.2 (1.8–14.1)	897	NA
Llama-4	25.0 (18.1–33.4)	25,136	28.8	30.0 (22.5–38.7)	17,055	28.9	10.3 (4.8–20.8)	2129	5.1
Original OpenManus	19.2 (13.1–27.1)	228,045	91.5	17.5 (11.7–25.3)	218,054	86.5	5.2 (1.8–14.1)	18,393	24.2
vOpenManus	27.5 (20.3–36.1)	206,124	88.9	29.2 (21.8–37.8)	209,535	71.1	15.5 (8.4–26.9)	20,022	13.4
Manus	NA	NA	NA	NA	NA	NA	5.2 (1.8–14.1)	NA	NA

Tokens refer to the average number of tokens consumed per scenario. Time refers to the average response time per scenario. Three agent systems were evaluated: the original OpenManus, OpenManus equipped with a medical-vision tool (vOpenManus), and Manus. Manus, a commercial closed-source system, cannot participate in interactive AgentClinic workflows. MedGemma failed to produce answers on the AgentClinic benchmark. Time measurements are available only for comparisons between OpenManus-based agent systems and their backbone model, Llama-4. Values in brackets denote 95% confidence intervals. NA indicates non-applicable data.

In HLE multimodal tasks, baseline LLM accuracies were 5.2% (Gemma-3 and MedGemma), 10.3% (GPT-4.1), and 10.3% (Llama-4). Both Manus and original OpenManus achieved 5.2% while vOpenManus improved accuracy to 15.5% (Fig. 3e, f). No statistically significant differences were observed among model performances (*p* = 0.213) (Fig. S2a).

Collectively, our findings indicate that agentic AI systems provide only modest improvements in accuracy relative to baseline LLMs, even after task-specific customization. Accuracy remained generally low across diverse clinical scenarios, challenging medical question answering, and multimodal diagnostic tasks.

### Agent systems substantially increase computational resources and response times

**Fig. 4:**
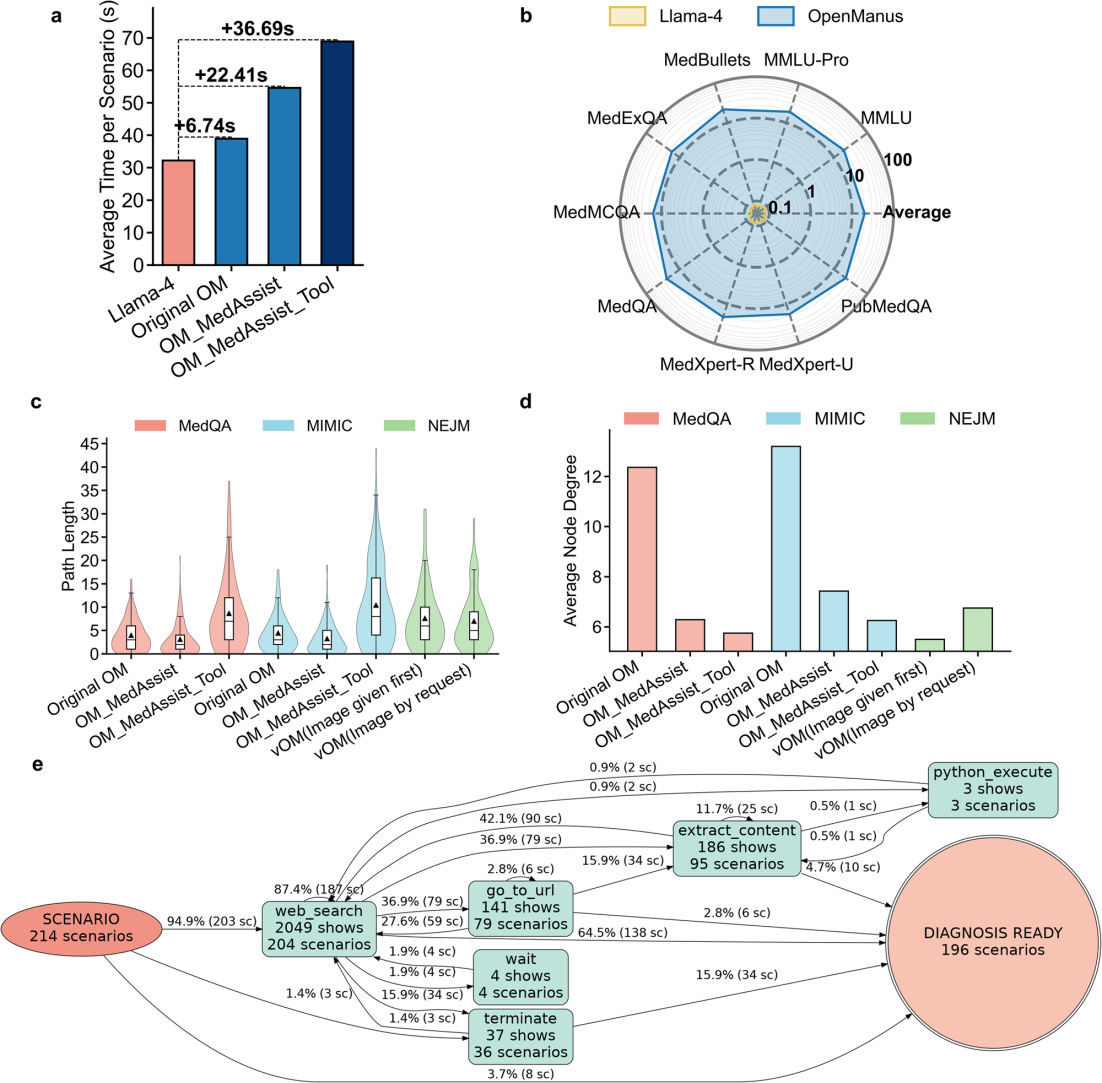
Efficiency and process metrics evaluation of OpenManus-based agent systems. **a** Average time consumption per scenario for the original OpenManus, OpenManus with a physician-assistant prompt (OM_MedAssist), and further encouraged to use tools (OM_MedAssist_Tool), compared with their backbone model Llama-4 on AgentClinic–MedQA. **b** Average time (second) consumption for OpenManus and its backbone Llama-4 across each dataset in MedAgentsBench, as well as the overall average. **c** Path length, defined as the total number of actions and tool invocations taken from scenario start to final decision. The violin plot depicts the distribution, the box plot shows the interquartile range and median, and the triangle marker denotes the mean. **d** Average node degree in the decision workflow graph, representing the mean number of connections per state; higher values indicate greater workflow complexity. **e** Workflow graph for OpenManus_MedAssist_Tool on AgentClinic–MedQA. Each node represents tool invocation or action; edges indicate execution order. OM OpenManus, sc scenario.

We next evaluated the computational efficiency of agent systems by assessing token usage and response times compared to baseline LLMs across multiple benchmarks. In the MedQA dataset of AgentClinic, token consumption per scenario was notably higher for OpenManus variants: original OpenManus utilized 92,954 tokens, OpenManus_MedAssist used 142,316, and OpenManus_MedAssist_Tool reached 168,957 tokens, compared to only 14,576 tokens for Llama-4 (Fig. 2b and Table 1). Regarding response time, Llama-4 took 32.2 s per scenario. In contrast, response times increased progressively for OpenManus variants: original OpenManus took 39.0 s, OpenManus_MedAssist required 54.6 s, and OpenManus_MedAssist_Tool averaged 68.9 s (Fig. 4a and Table 1). Similar patterns emerged with the MIMIC-IV dataset, where original OpenManus consumed up to 217,425 tokens per scenario, markedly higher than the 14,855 tokens used by Llama-4 (Fig. 2c and Table 1).

When evaluated on the MedAgentsBench, OpenManus consumed significantly more tokens per question (29,150 tokens) compared to Llama-4 (276 tokens) (Fig. 2e and Table 1). Response times also differed substantially, with OpenManus averaging 20.0 s per scenario versus just 0.08 s for Llama-4 (Fig. 4b and Table 1).

A similar trend was observed in the HLE benchmark, where OpenManus consumed an average of 14,837 tokens per question, markedly higher than the 1061 tokens used by Llama-4 (Fig. 3b). Correspondingly, response time increased from 7.0 s for Llama-4 to 11.3 s for OpenManus (Fig. S4b and Table 1).

In multimodal evaluations using the AgentClinic NEJM dataset, token usage was highest when images presented upfront: original OpenManus consumed 228,045 tokens, followed closely by vOpenManus with 206,124 tokens, both exceeding Llama-4’s 25,136 tokens per scenario (Fig. 3c and Table 1). Response times mirrored this trend, with original OpenManus taking 91.5 s and vOpenManus 88.9 s per scenario, substantially longer than Llama-4’s 28.8 s (Fig. S4c and Table 1). This pattern persisted when images were provided only upon request (Fig. S4d and Table 1).

Regarding multimodal tasks within the HLE dataset, token consumption per question increased dramatically from 2,129 tokens (Llama-4) to 20,022 tokens for vOpenManus (Fig. 3f and Table 1). Response times also increased considerably: Llama-4 averaged 5.1 s per scenario, while vOpenManus took 13.4 s, and original OpenManus required 24.2 s (Fig. S4e and Table 1).

Overall, these results highlight that the agent-based systems significantly elevate computational demands, consistently requiring more than tenfold the token usage and at least twice the response time compared to baseline LLMs.

### Task-customized agent systems reduced workflow complexity and improved tool usage consistency

To further explore agent system efficiency, we assessed the complexity of internal workflows by representing their processes as flowcharts, treating each tool call or action as a node, and quantifying complexity using graph metrics.

Original OpenManus demonstrated shorter paths but higher overall complexity across both MedQA and MIMIC-IV datasets, with average path lengths of 3.99 and 4.98, node degrees of 12.36 and 13.56, and cyclomatic complexities of 59 and 54, respectively (Figs. 4c, d, S4h and Table S1). In MedQA, nine distinct tools or actions were utilized, with frequent calls to web_search (264 calls across 106 scenarios) and extract_content (192 calls across 100 scenarios) (Fig. S5a). On average, 6.54 dialogs per scenario were required to reach a diagnosis (Fig. S4f and Table S1).

OpenManus_MedAssist, the physician-assistant customized variant, reduced overall workflow complexity, exhibiting lower node degrees (6.29 in MedQA, 7.43 in MIMIC-IV) and cyclomatic complexities (17 in MedQA, 21 in MIMIC-IV), while maintaining similar path lengths (3.09 in MedQA, 3.27 in MIMIC-IV) (Figs. 4c, d, S4h and Table S1). This variant showed increased web_search usage (499 calls across 154 scenarios in MedQA) relative to the original OpenManus (Fig. S5b).

Further refinement with OpenManus_MedAssist_Tool resulted in longer reasoning paths but further reduced complexity, achieving path lengths of 9.45 (MedQA) and 11.34 (MIMIC-IV), lower node degrees (5.75 MedQA, 6.25 MIMIC-IV), and reduced cyclomatic complexities (17 MedQA, 19 MIMIC-IV) (Figs. 4c, d, S4h and Table S1). Tool utilization significantly increased, particularly for web_search (2049 calls across 204 scenarios) and extract_content (186 calls across 95 scenarios in MedQA) (Fig. 4e). Consequently, dialogs required for diagnosis increased to an average of 9.23 (MedQA) and 11.66 (MIMIC-IV) per scenario (Fig. S4f and Table S1).

In multimodal scenarios with images provided upfront, the image_analysis tool was consistently employed in all scenarios alongside web_search, which was called 993 times across 110 scenarios (Fig. S5f). These workflows had an average path length of 7.58, a node degree of 5.5, and cyclomatic complexity of 16 (Figs. 4c, d, S4h and Table S1). When images were provided only upon request, the image_analysis tool was utilized in 83 scenarios (152 calls), with web_search used extensively (650 calls across 107 scenarios) (Fig. S5g). Here, the average path length was 6.98, with a node degree of 6.75, and cyclomatic complexity of 21 (Figs. 4c, d, S4h and Table S1).

We validated our local OpenManus implementation process on MedAgentBoard, achieving an overall correctness rate of 48%, comparable to the previously reported 50% (Fig. S6).

In summary, initial agent systems exhibited significant complexity due to diverse tool and action usage. Task-specific prompt engineering successfully streamlines workflows, reducing complexity by encouraging more targeted and consistent tool utilization.

### Hallucinations remain prevalent but effectively mitigated through targeted intervention

Considering patient safety as a crucial factor in medical applications, we conducted an analysis of hallucinations within OpenManus using the AgentClinic benchmarks. Specifically, we focused on two main hallucination types: fabricated patient statements and invented measurement results. We quantified their frequency, the number successfully blocked by the Llama-4 post-processing module, and the consequent impact on diagnostic performance.

**Fig. 5:**
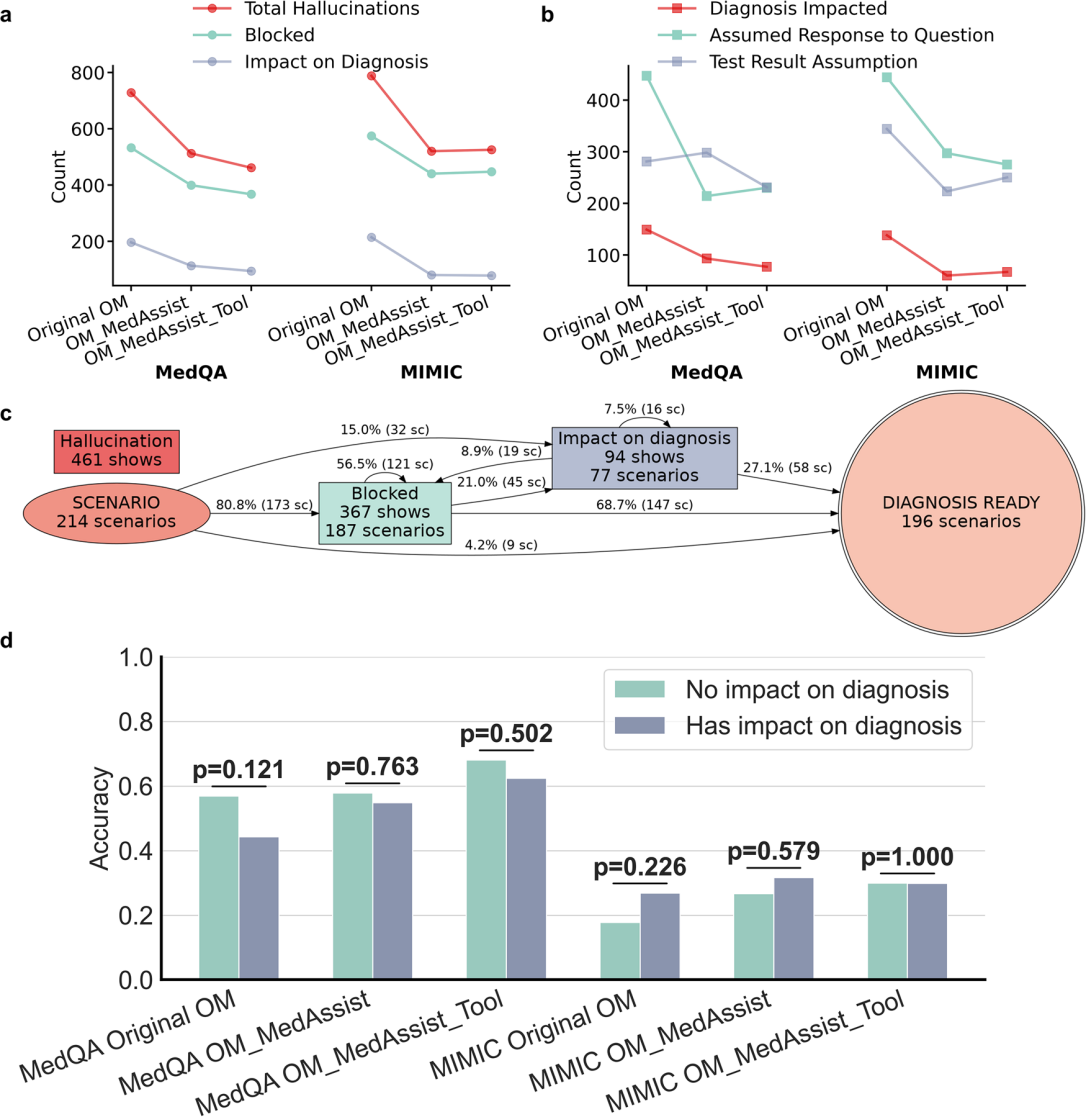
Hallucination analysis of OpenManus-based agent systems. **a** Total hallucination counts for each agent system on the AgentClinic–MedQA and AgentClinic–MIMIC datasets. “Blocked” refers to hallucinations successfully removed by the Llama-4 post-processing module, whereas “Impact on Diagnosis” refers to hallucinations that were not blocked and ultimately influenced the final diagnostic outcome. **b** Number of diagnoses impacted by hallucinations, along with counts of the two hallucination types. **c** Hallucination Maps of OpenManus_MedAssist_Tool on AgentClinic–MedQA. Nodes depict hallucination types and edges trace propagation. **d** Paired bars show model accuracy without and with diagnosis-impacting hallucinations; χ² *p*-values are annotated above each pair. OM OpenManus.

In the MedQA dataset, original OpenManus generated 728 hallucinations (mean 3.4 per scenario) across 208 scenarios (97.2%), blocking 532 and leaving 196 unblocked hallucinations affecting diagnoses in 149 scenarios (69.6%) (Figs. 5a, b, S7a, S8a and Table S2). OpenManus_MedAssist reduced hallucinations to 512 (mean 2.4 per scenario) across 193 scenarios (96.5%), blocking 399 and leaving diagnostic impact in 93 scenarios (43.5%) (Figs. 5a, b, S7b, S8a and Table S2). OpenManus_MedAssist_Tool produced 461 hallucinations (mean 2.2 per scenario) across 205 scenarios (95.8%), blocking 367 and affecting 77 scenarios (36.0%) (Figs. 5a–c, S8a and Table S2).

Similarly, in the MIMIC-IV dataset, original OpenManus generated 788 hallucinations (mean 3.9 per scenario) across 198 scenarios (99.0%), blocking 574 and affecting 138 scenarios (69.0%). OpenManus_MedAssist yielded 520 hallucinations (mean 2.6 per scenario) across 193 scenarios (96.5%), blocking 440 and affecting 60 scenarios (30.0%). OpenManus_MedAssist_Tool produced 525 hallucinations (mean 2.6 per scenario) across 197 scenarios (98.5%), blocking 447 and affecting 67 scenarios (33.5%) (Figs. 5a, b, S7c–e, S8b and Table S2).

Overall, interventions including LLM-based output filtering, targeted prompt engineering, and few-shot learning effectively resolved 89.9% of hallucinations, significantly reducing the diagnostic risk. Among the 1516 hallucinations initially generated by original OpenManus, the refined OpenManus_MedAssist_Tool reduced diagnostically relevant hallucinations to 155. Notably, there was no statistically significant difference in diagnostic accuracy between scenarios with hallucinations and those without (Fig. 5d).

In summary, hallucinations were widespread across all agent system variants; however, targeted methodological improvements substantially mitigated their impact, highlighting the effectiveness of output filtering, prompt engineering, and task-specific customization strategies.

## Discussion

This study represents a systematic evaluation on the performance of LLM-based agent systems compared to baseline state-of-the-art LLMs across various healthcare-related benchmarks, including diagnostic dialogs, complex medical questions, and multimodal tasks.

Our key findings indicate that task-specific customization through prompt engineering and tool augmentation of agent systems resulted in modest accuracy improvements over baseline LLMs, with absolute accuracy gains ranging between 7.0% and 8.9% in LLM-simulated diagnostic dialogs (MedQA and MIMIC-IV). On single-turn medical question-answering tasks (MedAgentsBench and HLE), OpenManus also achieved small improvements. However, these improvements did not translate into overall high accuracy and were consistently accompanied by increased computational resource demands, longer response times, and persistent hallucinations, underscoring significant practical trade-offs.

Overall, the agent systems achieved the highest accuracies across all text-only benchmarks; however, their advantage over the best-performing baseline LLMs was modest—ranging from 0.5 to 8.9%—and did not reach statistical significance. When comparing performance improvements against computational costs, our findings reveal a clear imbalance. Across all benchmarks, OpenManus variants consumed 10–100 times the tokens of their backbone Llama-4 model and frequently doubled or tripled the response time. These results suggest that while agent systems have the potential to enhance clinical decision-making accuracy, especially through structured tool integration, they remain limited by inefficiency and inherent complexity. This aligns with previous research documenting the resource-intensive nature of agent systems^22^. Such inefficiencies could pose considerable barriers to their deployment in real-world clinical settings, particularly where rapid and resource-efficient decision-making is essential.

The observed modest accuracy gains can largely be attributed to the structured hierarchical design and systematic integration of external tools within agent architectures, enabling access to timely medical information. The structured use of web browsing and other external tools, particularly noted in OpenManus variants, aligns with previous studies highlighting performance gains when external resources are incorporated into medical reasoning tasks^23,24^. In contrast, baseline models such as GPT-4.1 demonstrated significant restrictions due to internal safety constraints, and models such as Qwen-3 frequently produced inaccurate diagnostic reasoning due to premature conclusions, likely stemming from defective chain-of-thought generation^25^. The medically fine-tuned model also performed poorly, likely due to its relatively small size and narrow training domain, which limit its ability to generalize across diverse tasks.

The consistency of our results with prior research further reinforces the existing understanding in this domain. For example, previous work has shown structured agent architectures, such as the multi-persona debate model by Microsoft and the conversational multi-LLM framework proposed by Chen et al., effectively enhance diagnostic accuracy through the role specialization^26,27^. Similarly, our task-specific customization significantly reduced workflow complexity, enhancing clarity and diagnostic performance, demonstrating the importance of well-defined agent roles and structured prompting.

Nevertheless, the multimodal performance of agent systems did not consistently improve over baseline models. We attribute this inconsistency primarily to suboptimal medical image analysis tools and internal tool competition, highlighting a critical area for future development. Additionally, single-turn tasks showed more pronounced benefits from agent systems, indicating their particular suitability for specific clinical question-answering contexts compared to more complex dialog scenarios.

Persistent hallucinations also emerged as a critical concern. Previous research has suggested that hallucination is an inherent characteristic of language generation based on LLMs^28^, and agent systems may amplify these effects through repeated LLM calls. Despite successful mitigation strategies (output filtering, prompt engineering, and few-shot learning), hallucinations continued to affect approximately 30% of clinical scenarios. Importantly, our analysis revealed no significant accuracy differences between cases impacted and unaffected by hallucinations, possibly because hallucinations prompted further information gathering, indirectly benefiting clinical reasoning.

This study has considerable implications for the future development of agentic AI clinical decision support systems. Interest in agentic AI has risen dramatically in 2025, driven by expectations that multi-agent systems will achieve greater capability than single LLMs^29^. As this is still a new research area with limited clinical guidance, our analysis suggests that development should proceed with careful evaluation rather than assumptions of automatic superiority. Real-world clinical use requires multidimensional assessment across accuracy, efficiency, and safety, each essential for clinical decision-making. However, current studies tend to increase architectural complexity, demonstrate strong results only in narrow scenarios, and lack comprehensive assessment or real-world validation^30^. Guidelines on clearer research frameworks and evaluation standards to support systematic design and clinical application are required. Future progress may benefit from prioritizing accuracy, efficiency, hallucination control, and stronger medical grounding under human supervision, validated on comprehensive benchmarks containing large real-world cohorts, ensuring that advancement aligns with clinical needs and safety requirements before real-world deployment.

This study has several important limitations that constrain the direct clinical applicability of our findings. First, all the benchmarks represent idealized and highly structured task formulations that only approximate real-world clinical encounters. Real-world encounters involve multiple concurrent complaints, evolving symptoms, incomplete or contradictory information, communication ambiguity, and substantial patient heterogeneity—complexities not fully captured by our benchmarks. As a result, the observed performance of agent systems may overestimate their reliability in unstructured or atypical clinical settings. Second, our evaluations do not cover longitudinal disease trajectories, rich multimorbidity patterns, or the full diversity of clinical imaging modalities. Thus, the generalization of agentic reasoning to broader clinical contexts remains uncertain. Third, safety assessments were conducted in controlled simulations. Real-world deployment would require prospective validation, robust safety mechanisms, and regulatory oversight, which were beyond the scope of this study. Finally, the closed-source nature of some systems, such as Manus, limited our ability to analyze internal reasoning or failure modes. Future work should incorporate authentic EHR data, more complex clinical scenarios, and longitudinal workflows to better approximate real-world clinical complexity.

In conclusion, while LLM-based agent systems offer promising improvements in accuracy through structured customization and external tool integration, they currently remain insufficiently robust, efficient, and reliable for routine clinical deployment. Continued refinement addressing identified limitations and trade-offs is essential to realize the full potential of agentic AI in healthcare settings.

## Methods

### Benchmarks and datasets

AgentClinic: AgentClinic is an open-source, multimodal benchmark that adapts the objective-structured clinical examinations (OSCEs) format to a multi-agent, dialog-based simulation of real-world diagnostic encounters using LLM-generated interactions^10^. Four agents–doctor, patient, measurement, and moderator–engage in up to 20 inference turns, after which the doctor must output a final diagnosis as a free-text string. In all experiments, the doctor agent was powered by the model under evaluation, whereas the patient, measurement, and moderator roles ran on GPT-4o-mini due to their simpler roles.

Three AgentClinic subsets were evaluated. AgentClinic-MedQA contains 214 scenarios from the MedQA board-exam corpus^31^; each providing structured facts, such as objective, history, symptoms, laboratories, and a gold-standard diagnosis. AgentClinic-MIMIC-IV comprises 200 de-identified inpatient encounters from the MIMIC-IV EHR database^32,33^, adapted into an OSCE format using Llama-4 and manual verification, each bearing a unique diagnosis. AgentClinic-NEJM features 120 image-centric diagnostic challenges from the *New England Journal of Medicine* case-challenge series, pairing at least one radiograph or clinical photograph with clinical history to demand combined visual-textual reasoning.

MedAgentsBench: MedAgentsBench targets complex medical question-answering tasks which remain difficult for current LLMs, with GPT-4o-mini and GPT-4o achieving average accuracies of only 10.8 and 18%, respectively^12^. We adopted the publicly released HARD subset (862 multiple-choice questions), created with adversarial filtering to retain only items answered correctly by <50% of a diverse LLM panel. The subset maintains domain balance by sampling: 100 questions each from MedQA^31^, PubMedQA^34^, MedMCQA^35^, MedExQA^36^, and MMLU-Pro^37^, 89 from MedBullets^38^, 73 from MMLU^39^, and 100 each from the Reasoning and Understanding splits of MedXpertQA^40^. This offers a uniform evaluation set for diagnosis and treatment planning questions that would otherwise show ceiling effects on standard benchmarks.

Humanity’s Last Exam (HLE): HLE is a multi-disciplinary benchmark of highly complex and nuanced multiple-choice and short-answer questions designed to defeat shortcut cues^41^. We evaluated the Biology/Medicine subset, comprising 222 text-only tasks and 58 multimodal items.

### Baseline LLMs

For text-only benchmarks we evaluated Meta’s Llama-4-Maverick-17B-128E-Instruct-FP8, Ali’s Qwen-3-235B-A22B-FP8, Google’s MedGemma-27B-IT-FP8 (a medical-domain fine-tuned model based on Gemma-3) and OpenAI’s GPT-4.1; for multimodal tasks we used the same Llama-4 configuration, Google’s Gemma-3-27B-IT-Q8 and MedGemma-27B-IT-FP8, and GPT-4.1. All models were run zero-shot. Llama-4 and Qwen-3 were hosted locally via vLLM^42^ on a four and two NVIDIAH200 GPUs, respectively. MedGemma were hosted via vLLM^42^ on a single A6000 GPU. Gemma-3 used llama.cpp^43^ on a single A6000 GPU; GPT-4.1 was accessed through the OpenAI API at https://api.openai.com/v1^44^.

### Agent systems

Manus is a closed-source, commercial multi-agent framework that runs a planner-executor-verifier loop and calls external tools such as a browser, code runner, and file editor^19^. Since Manus cannot be embedded into AgentClinic, we evaluated it only on MedAgentsBench and HLE via its public web interface, recording the returned answers.

OpenManus is an MIT-licensed, open-source implementation of Manus that organizes agents in a three-layer hierarchy (BaseAgent, ReActAgent, and ToolCallAgent) and supplies built-in tools for web browsing, code execution, and text editing^45^. Every variant evaluated in our study used the same Llama-4 backbone to isolate behavioral differences. Task-specific customizations were introduced through prompt engineering and tool augmentation. We tested (i) the unmodified codebase (Original), (ii) a prompt-engineered *MedAssist* version that frames the system as a physician assistant and adds prompts and few-shot exemplars to restrict hallucinations, available for AgentClinic tasks (iii) a prompt-engineered *MedAssist_Tool version,* which further encourages tool use when clinical uncertainty arises, available for AgentClinic tasks, and (iv) *vOpenManus*, which incorporates a custom medical-vision tool for multimodal AgentClinic and HLE tasks while otherwise mirroring MedAssist-Tool. In AgentClinic, a lightweight Llama-4 post-processor converted agent outputs to the required dialog format and filtered hallucinated content; in MedAgentsBench, a GPT-4o-mini post-processor compressed answers to single-letter choices. Implementation validation was verified on the MedAgentBoard benchmark^46^, where our local run matched the reported top performance on the Tongji Hospital dataset.

### Endpoints and metrics

The primary endpoint was accuracy, defined as the proportion of model outputs that matched ground-truth labels. Secondary endpoints targeted practical cost and risk. Efficiency was measured as total tokens consumed and time-on-task. Workflow complexity (AgentClinic) was assessed by modeling each tool call or action as a node in a flowchart and computing mean and maximum path length, average node degree, and cyclomatic complexity (*E* − *N* + 2*P*, where *E* is edges, *N* is nodes, and *P* is connected components) to quantify search depth and branching. Safety (AgentClinic) was evaluated by assessing hallucinations. Hallucinations were identified by manual review of agents’ reasoning and dialog content against patient records. By assessing the reasoning steps prior to the post-processor, two types of hallucination were quantified: 1. Assumed response to question: the agent posed a question to the patient and subsequently assumed a response during later reasoning steps, despite no corresponding real patient reply. 2. Test result assumption: the agent generated test or laboratory values without requesting such data from the patient record. Each hallucination was then classified as either: 1. Blocked: in the dialog, the hallucination appeared, but the doctor’s words to the patient did not treat the hallucination as an established fact. 2. Impacted on diagnosis: any hallucination that did not meet criterion 1. A scenario was considered hallucination-impacted if it contained at least one hallucination categorized as impacted on diagnosis.

### Statistical analysis

Statistical analyses were performed using Python (version 3.12). Overall differences in accuracy among models for each benchmark were assessed using Cochran’s Q test. McNemar’s test was used for pairwise comparisons of model accuracies, with *p* values Holm-adjusted to control for multiple comparisons. 95% confidence intervals were calculated using the Wilson score method. Chi-square tests were used to compare accuracy between hallucination-impacted and non-impacted cases. For multiple-choice datasets, chi-square goodness-of-fit tests were applied to evaluate whether the distribution of answer choices deviated from uniformity. A two-sided *p* < 0.05 was considered statistically significant.

### Ethics statement

This study does not include confidential information. All research procedures were conducted exclusively on publicly accessible and de-identified data in accordance with the Declaration of Helsinki, maintaining all relevant ethical standards. The overall analysis was approved by the Ethics Commission of the Medical Faculty of the Technical University Dresden (BO-EK-444102022).

## Supplementary information

Supplementary Information

## Data Availability

The MIMIC-IV dataset can be accessed at https://physionet.org/content/mimiciv/3.1/ upon submission and approval of a data access application. All other data are available at https://github.com/NCCYUNSONG/AgentBenchMedicine_source.

## Code availability

All source codes are available at https://github.com/NCCYUNSONG/AgentBenchMedicine_source.
