# Supplementary material for: Benchmarking large language model-based agent systems for clinical decision tasks
Source: NPJ Digit Med. 2026 Feb 18;9:259. doi: 10.1038/s41746-026-02443-6 (PMC13031301; doi:10.1038/s41746-026-02443-6)
Supplement: Supplementary file 1 — Supplementary Information [file 41746_2026_2443_MOESM1_ESM.pdf]

Benchmarking Large Language Model-Based Agent Systems for Clinical Decision Tasks

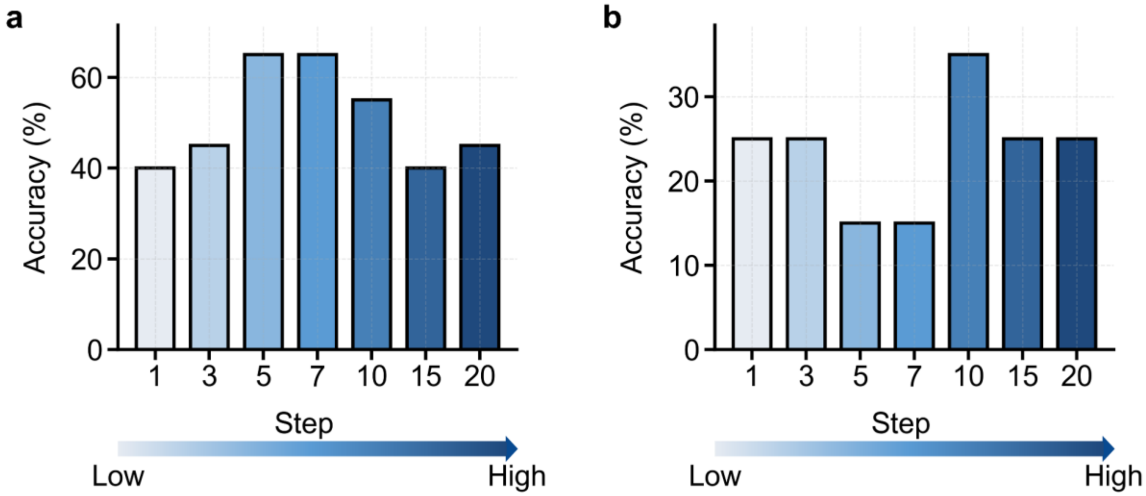

Figure S1. Effect of step limit. Accuracy changes as the step limit increases in a 20-scenario pilot on **a.** AgentClinic–MedQA, used to determine the step limit for AgentClinic tasks, and **b.** MedAgentsBench for single-round question-answering tasks. Step limits were selected based on achieving relatively best performance within a generally stable performance range.

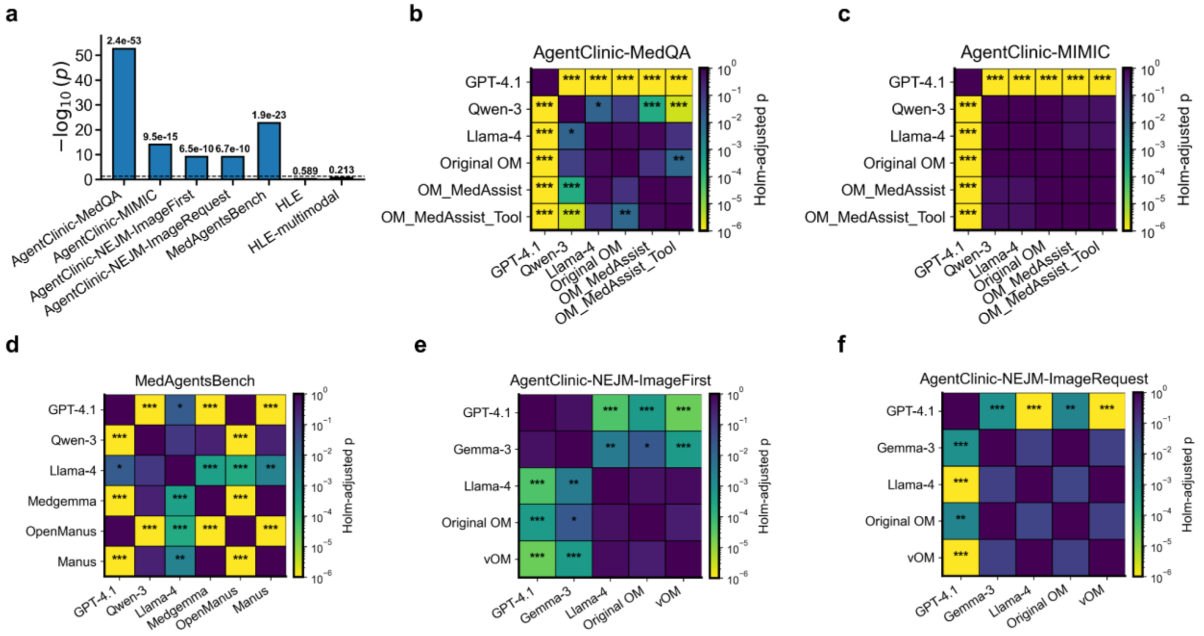

Figure S2. Significance testing for LLMs and agent systems. **a**. Cochran's Q test results across all benchmarks (dashed line indicates the 0.05 significance threshold). For benchmarks showing significant differences under Cochran's Q, Holm-adjusted pairwise McNemar tests were conducted for **b**. AgentClinic–MedQA, **c**. AgentClinic–MIMIC, **d**. MedAgentsBench, **e**. AgentClinic–NEJM with images provided upfront, and **f**. AgentClinic–NEJM with images provided only upon request. McNemar p-values were Holm-adjusted for multiple comparisons. Significance levels: * ≤ 0.05; ** ≤ 0.01; *** ≤ 0.001. OM, OpenManus.

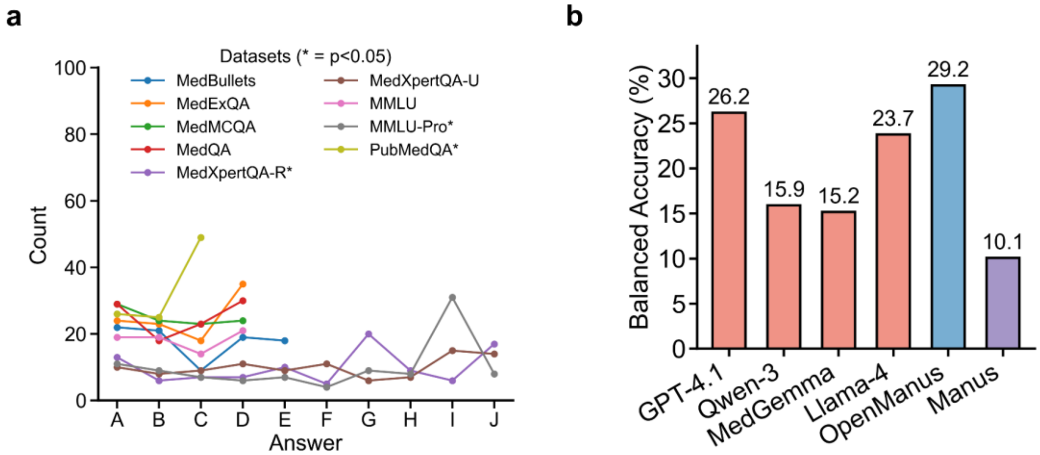

Figure S3. Impact of answer-choice distribution in MedAgentsBench. **a**. Distribution of predicted answer choices for each dataset in MedAgentsBench. *p*-values are calculated using a chi-square goodness-of-fit test to assess whether the observed choice distribution deviates from a uniform distribution. **b**. Balanced accuracy for each model, accounting for the underlying answer-choice distribution.

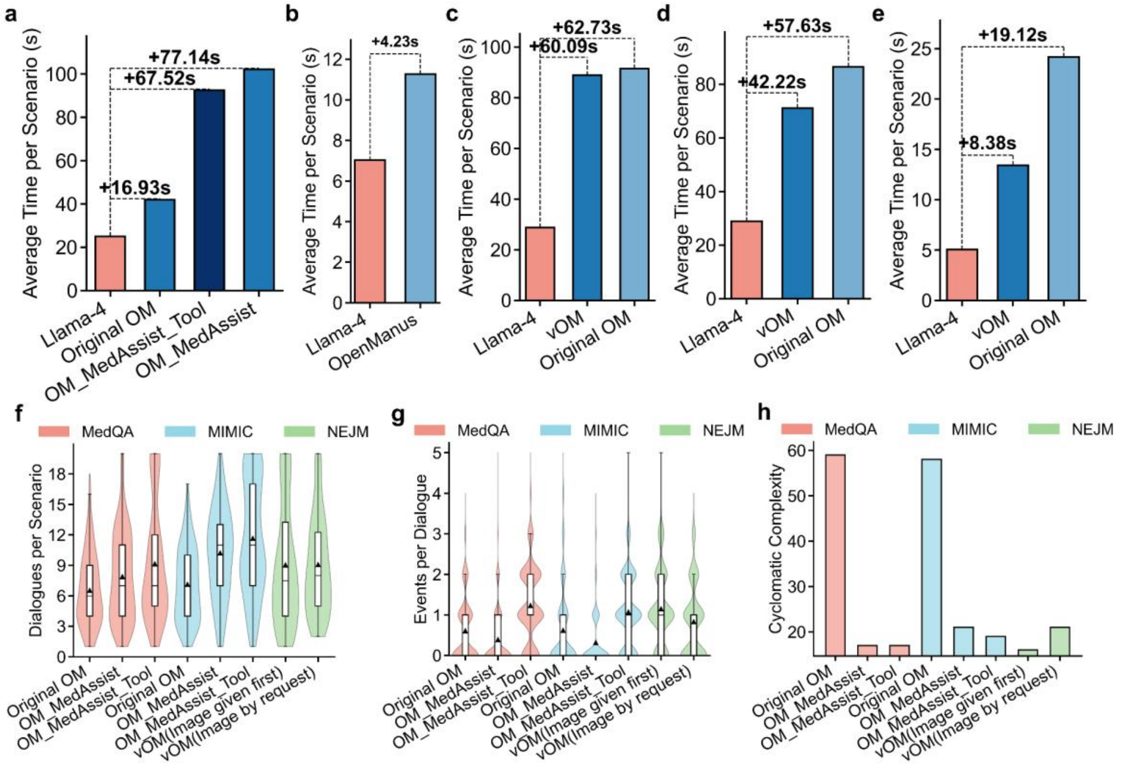

Figure S4. Additional process metrics evaluation of OpenManus-based agent systems. **a–e.** Average time consumption per scenario for OpenManus-based agent systems compared with their backbone model, Llama-4, across: **a**. AgentClinic–MIMIC, **b**. Humanity's Last Exam (HLE) text-only tasks, **c**. AgentClinic–NEJM multimodal tasks with images provided first, **d**. AgentClinic–NEJM multimodal tasks with images provided only upon request, and **e**. HLE multimodal tasks. **f.** Number of dialogues per scenario in AgentClinic tasks to reach a final diagnosis. **g.** Number of events per scenario in AgentClinic tasks, defined as the total actions or tool invocations per dialogue. **h.** Cyclomatic complexity of the workflow graph for AgentClinic tasks, computed as $E - N + 2P$, where E is the number of edges, N the number of nodes, and P the number of connected components.

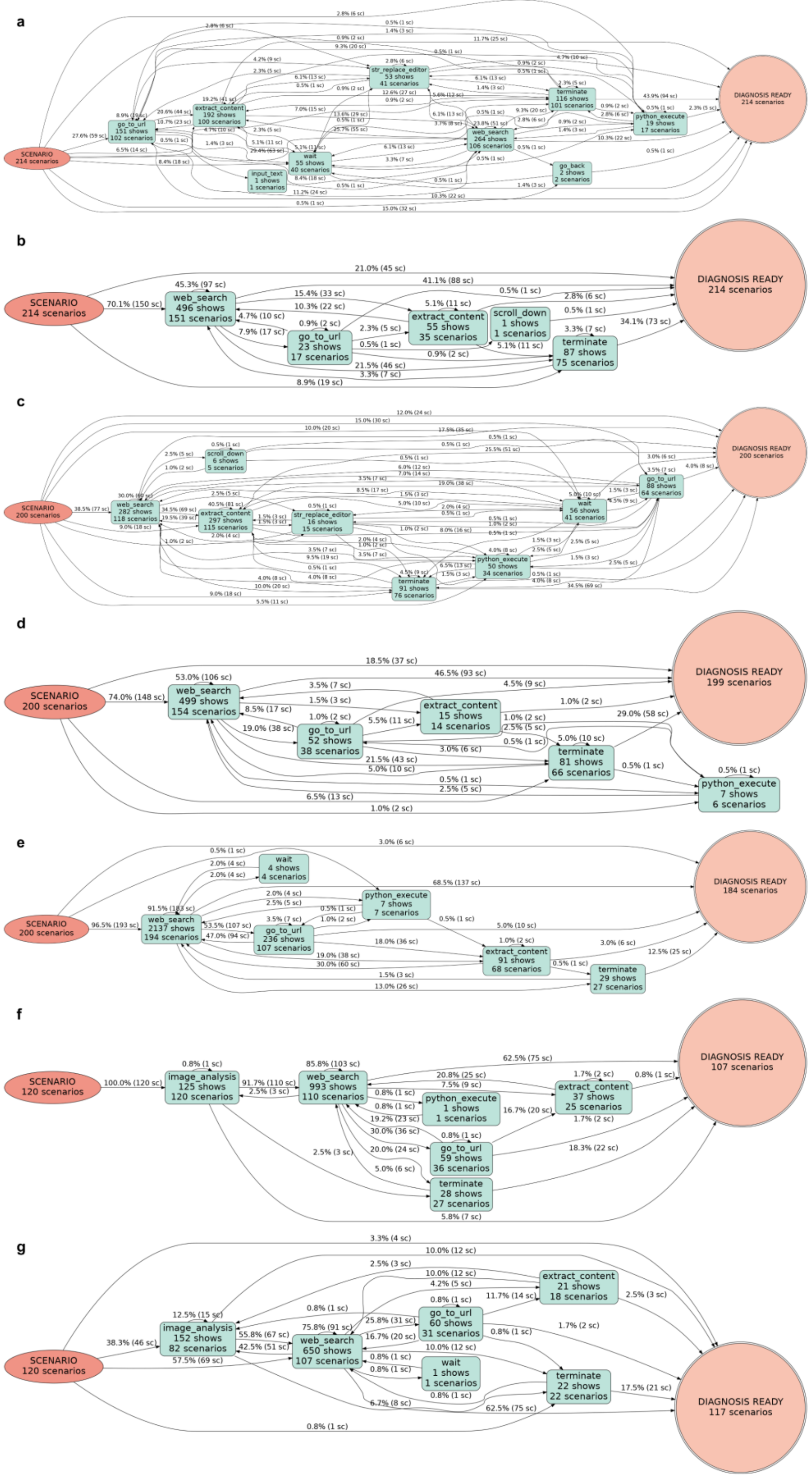
a
SCENARIO
214 scenarios
go_to_url
151 shows
102 scenarios
extract_content
192 shows
100 scenarios
str_replace_editor
53 shows
41 scenarios
terminate
116 shows
101 scenarios
python_execute
19 shows
17 scenarios
web_search
264 shows
106 scenarios
wait
55 shows
40 scenarios
input_text
1 shows
1 scenarios
go_back
2 shows
2 scenarios
DIAGNOSIS READY
214 scenarios
b
SCENARIO
214 scenarios
70.1% (150 sc)
web_search
496 shows
151 scenarios
45.3% (97 sc)
21.0% (45 sc)
41.1% (88 sc)
15.4% (33 sc)
10.3% (22 sc)
4.7% (10 sc)
7.9% (17 sc)
extract_content
55 shows
35 scenarios
5.1% (11 sc)
scroll_down
1 shows
1 scenarios
0.5% (1 sc)
2.8% (6 sc)
0.5% (1 sc)
go_to_url
23 shows
17 scenarios
0.9% (2 sc)
2.3% (5 sc)
0.5% (1 sc)
0.9% (2 sc)
3.3% (7 sc)
5.1% (11 sc)
34.1% (73 sc)
terminate
87 shows
75 scenarios
21.5% (46 sc)
3.3% (7 sc)
8.9% (19 sc)
DIAGNOSIS READY
214 scenarios
c
SCENARIO
200 scenarios
web_search
282 shows
118 scenarios
scroll_down
6 shows
5 scenarios
extract_content
297 shows
115 scenarios
str_replace_editor
16 shows
15 scenarios
wait
56 shows
41 scenarios
go_to_url
88 shows
64 scenarios
python_execute
50 shows
34 scenarios
terminate
91 shows
76 scenarios
DIAGNOSIS READY
200 scenarios
d
SCENARIO
200 scenarios
74.0% (148 sc)
web_search
499 shows
154 scenarios
53.0% (106 sc)
18.5% (37 sc)
46.5% (93 sc)
3.5% (7 sc)
4.5% (9 sc)
1.5% (3 sc)
8.5% (17 sc)
extract_content
15 shows
14 scenarios
1.0% (2 sc)
1.0% (2 sc)
5.5% (11 sc)
19.0% (38 sc)
go_to_url
52 shows
38 scenarios
1.0% (2 sc)
2.5% (5 sc)
29.0% (58 sc)
5.0% (10 sc)
3.0% (6 sc)
0.5% (1 sc)
terminate
81 shows
66 scenarios
21.5% (43 sc)
5.0% (10 sc)
0.5% (1 sc)
0.5% (1 sc)
2.5% (5 sc)
6.5% (13 sc)
1.0% (2 sc)
0.5% (1 sc)
python_execute
7 shows
6 scenarios
DIAGNOSIS READY
199 scenarios
e
SCENARIO
200 scenarios
96.5% (193 sc)
web_search
2137 shows
194 scenarios
91.5% (183 sc)
0.5% (1 sc)
2.0% (4 sc)
2.0% (4 sc)
wait
4 shows
4 scenarios
3.0% (6 sc)
python_execute
7 shows
7 scenarios
2.0% (4 sc)
2.5% (5 sc)
68.5% (137 sc)
53.5% (107 sc)
47.0% (94 sc)
3.5% (7 sc)
0.5% (1 sc)
1.0% (2 sc)
go_to_url
236 shows
107 scenarios
0.5% (1 sc)
5.0% (10 sc)
18.0% (36 sc)
1.0% (2 sc)
19.0% (38 sc)
30.0% (60 sc)
extract_content
91 shows
68 scenarios
3.0% (6 sc)
0.5% (1 sc)
12.5% (25 sc)
1.5% (3 sc)
13.0% (26 sc)
terminate
29 shows
27 scenarios
DIAGNOSIS READY
184 scenarios
f
SCENARIO
120 scenarios
100.0% (120 sc)
image_analysis
125 shows
120 scenarios
0.8% (1 sc)
91.7% (110 sc)
2.5% (3 sc)
web_search
993 shows
110 scenarios
85.8% (103 sc)
62.5% (75 sc)
20.8% (25 sc)
7.5% (9 sc)
1.7% (2 sc)
0.8% (1 sc)
extract_content
37 shows
25 scenarios
0.8% (1 sc)
0.8% (1 sc)
python_execute
1 shows
1 scenarios
19.2% (23 sc)
16.7% (20 sc)
1.7% (2 sc)
30.0% (36 sc)
0.8% (1 sc)
20.0% (24 sc)
go_to_url
59 shows
36 scenarios
18.3% (22 sc)
2.5% (3 sc)
5.0% (6 sc)
terminate
28 shows
27 scenarios
5.8% (7 sc)
DIAGNOSIS READY
107 scenarios
g
SCENARIO
120 scenarios
3.3% (4 sc)
10.0% (12 sc)
2.5% (3 sc)
10.0% (12 sc)
extract_content
21 shows
18 scenarios
4.2% (5 sc)
2.5% (3 sc)
11.7% (14 sc)
0.8% (1 sc)
12.5% (15 sc)
0.8% (1 sc)
go_to_url
60 shows
31 scenarios
1.7% (2 sc)
38.3% (46 sc)
image_analysis
152 shows
82 scenarios
55.8% (67 sc)
75.8% (91 sc)
25.8% (31 sc)
16.7% (20 sc)
0.8% (1 sc)
42.5% (51 sc)
web_search
650 shows
107 scenarios
10.0% (12 sc)
57.5% (69 sc)
0.8% (1 sc)
0.8% (1 sc)
wait
1 shows
1 scenarios
terminate
22 shows
22 scenarios
0.8% (1 sc)
17.5% (21 sc)
6.7% (8 sc)
62.5% (75 sc)
0.8% (1 sc)
DIAGNOSIS READY
117 scenarios

Figure S5. Workflow graph across AgentClinic datasets. **a**. Original OpenManus on AgentClinic–MedQA. **b**. OpenManus_MedAssist on AgentClinic–MedQA. **c**. Original OpenManus on AgentClinic–MIMIC. **d**. OpenManus_Med_Assist on AgentClinic–MIMIC. **e**. OpenManus_MedAssist_Tool on AgentClinic–MIMIC. **f**.vOpenManus when images are provided first in multimodal AgentClinic–NEJM. **g**. vOpenManus when images were given by request in multimodal AgentClinic–NEJM. Each node represents tool invocation or action; edges indicate execution order. sc, scenario.

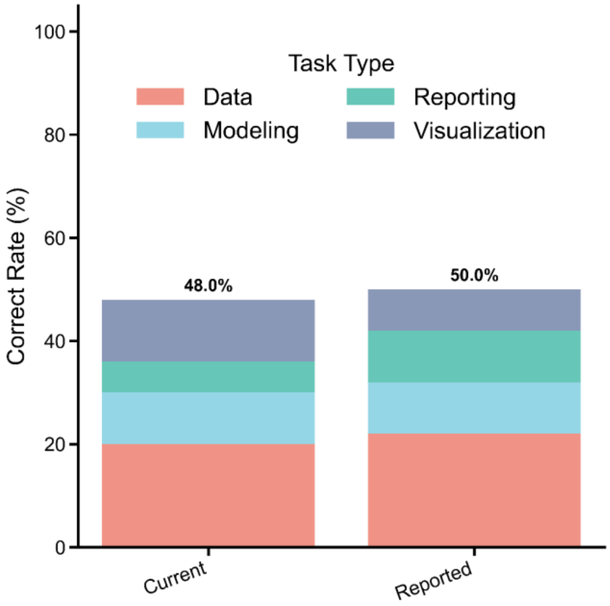

Figure S6. Validation results of local OpenManus implementation versus reported performance on MedAgentBoard.

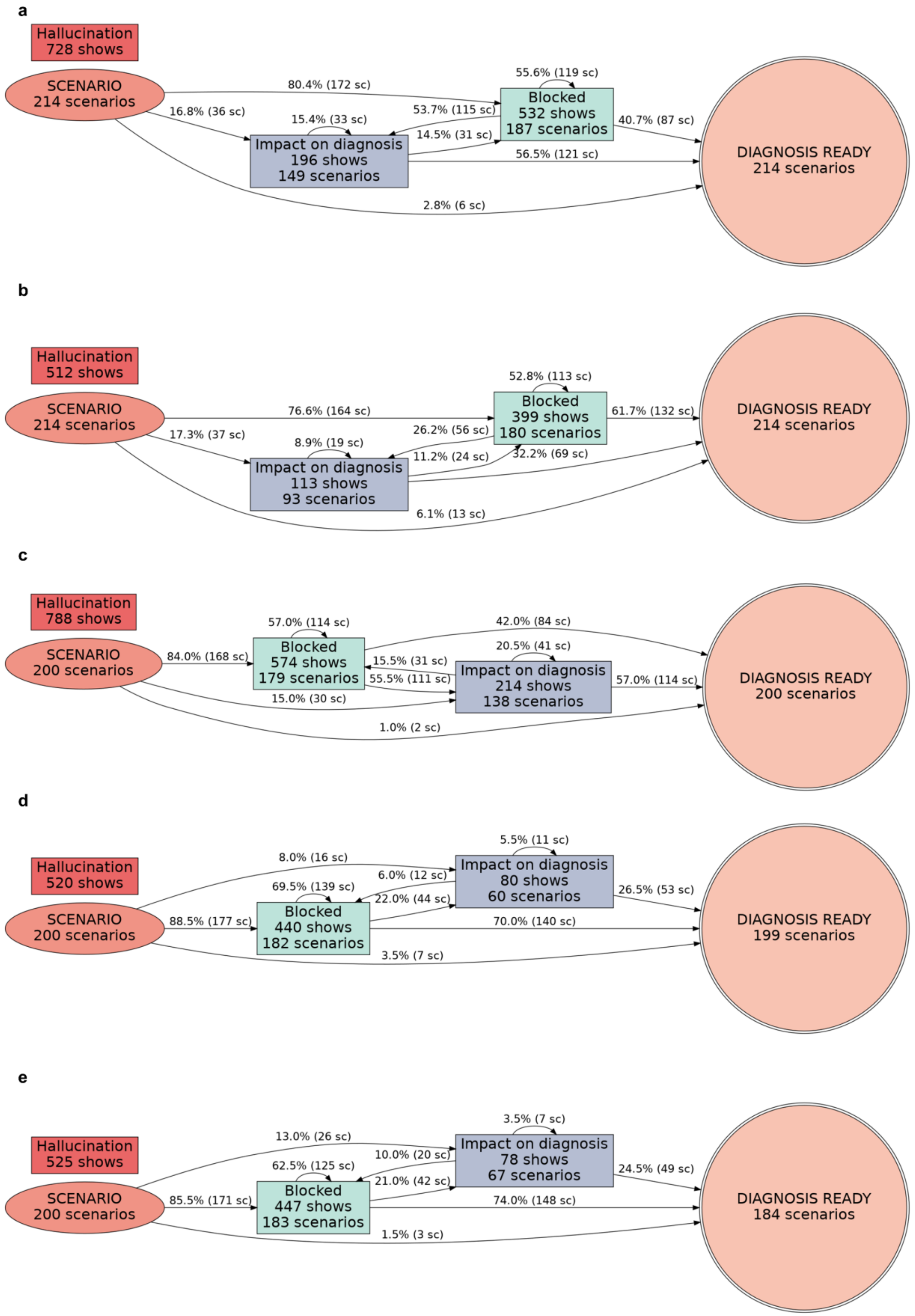
a
Hallucination
728 shows
SCENARIO
214 scenarios
80.4% (172 sc)
16.8% (36 sc)
55.6% (119 sc)
Blocked
532 shows
187 scenarios
53.7% (115 sc)
15.4% (33 sc)
14.5% (31 sc)
40.7% (87 sc)
Impact on diagnosis
196 shows
149 scenarios
56.5% (121 sc)
DIAGNOSIS READY
214 scenarios
2.8% (6 sc)
b
Hallucination
512 shows
SCENARIO
214 scenarios
52.8% (113 sc)
Blocked
399 shows
180 scenarios
76.6% (164 sc)
61.7% (132 sc)
DIAGNOSIS READY
214 scenarios
17.3% (37 sc)
26.2% (56 sc)
8.9% (19 sc)
11.2% (24 sc)
32.2% (69 sc)
Impact on diagnosis
113 shows
93 scenarios
6.1% (13 sc)
c
Hallucination
788 shows
57.0% (114 sc)
42.0% (84 sc)
SCENARIO
200 scenarios
84.0% (168 sc)
Blocked
574 shows
179 scenarios
20.5% (41 sc)
15.5% (31 sc)
55.5% (111 sc)
Impact on diagnosis
214 shows
138 scenarios
57.0% (114 sc)
DIAGNOSIS READY
200 scenarios
15.0% (30 sc)
1.0% (2 sc)
d
5.5% (11 sc)
8.0% (16 sc)
Impact on diagnosis
80 shows
60 scenarios
Hallucination
520 shows
69.5% (139 sc)
6.0% (12 sc)
22.0% (44 sc)
26.5% (53 sc)
SCENARIO
200 scenarios
88.5% (177 sc)
Blocked
440 shows
182 scenarios
70.0% (140 sc)
DIAGNOSIS READY
199 scenarios
3.5% (7 sc)
e
3.5% (7 sc)
13.0% (26 sc)
Impact on diagnosis
78 shows
67 scenarios
Hallucination
525 shows
10.0% (20 sc)
62.5% (125 sc)
21.0% (42 sc)
24.5% (49 sc)
SCENARIO
200 scenarios
85.5% (171 sc)
Blocked
447 shows
183 scenarios
74.0% (148 sc)
DIAGNOSIS READY
184 scenarios
1.5% (3 sc)

Figure S7. Hallucination maps across AgentClinic datasets. Nodes depict hallucination types and edges trace propagation: a. Original OpenManus on AgentClinic–MedQA. b. OpenManus_MedAssist on AgentClinic–MedQA. c. Original OpenManus on AgentClinic–MIMIC. d. OpenManus_MedAssist on AgentClinic–MIMIC. e. OpenManus_MedAssist_Tool on AgentClinic–MIMIC. sc, scenario.

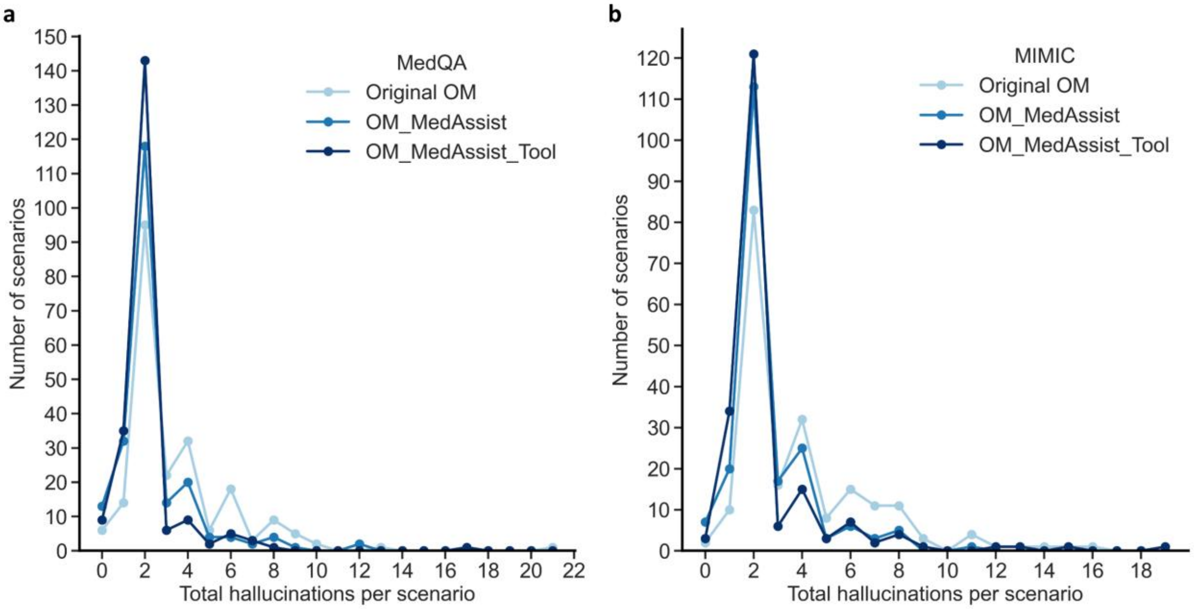

Figure S8. Distribution of hallucinations per scenario prior to blocking across AgentClinic datasets. a, MedQA. b, MIMIC. OM, OpenManus.

Table S1. Workflow Metrics

Metrics	MedQA			MIMIC			NEJM	
	Original OpenManus	OpenManus_MedAssist	OpenManus_MedAssist_Tool	Original OpenManus	OpenManus_MedAssist	OpenManus_MedAssist_Tool	vOpenManus (Image given First)	vOpenManus (Image by request)
Average Path Length	3.99	3.09	9.45	4.98	3.27	11.34	7.58	6.98
Maximum Path Length	16	21	37	20	19	44	31	29
Average Node Degree	12.36	6.29	5.75	13.56	7.43	6.25	5.50	6.75
Cyclomatic Complexity	59	17	17	54	21	19	16	21
Total Events	853	662	2420	996	654	2504	1243	906
Average Dialogues per	6.54	7.89	9.23	7.13	10.21	11.66	9.04	9.07

Scenario								
Average Events per Dialogue	0.61	0.39	1.23	0.62	0.32	1.07	1.15	0.83

Events are defined as tools or actions taken by OpenManus. Path Length represents the number of events from scenario start to final decision. Average Node Degree represents the average number of connections per state in the decision workflow graph. Cyclomatic complexity of the workflow graph for AgentClinic tasks, computed as $E - N + 2P$, where E is the number of edges, N the number of nodes, and P the number of connected components.

Table S2. Hallucination Analysis of OpenManus

Index	MedQA			MIMIC		
	Original OpenManus	OpenManus_MedAssist	OpenManus_MedAssist_Tool	Original OpenManus	OpenManus_MedAssist	OpenManus_MedAssist_Tool
Total Hallucinations prior to blocking	728	512	461	788	520	525
Average Hallucinations per scenario prior to blocking	3.4	2.4	2.2	3.9	2.6	2.6

Scenarios containing hallucination prior to blocking	208 (97.2%)	201 (93.9%)	205 (95.8%)	198 (99.0%)	193 (96.5%)	197 (98.5%)
Blocked	532	399	367	574	440	447
Impact on Diagnosis	196	113	94	214	80	78
Scenarios with Impact on Diagnosis	149 (69.6%)	93 (43.5%)	77 (36.0%)	138 (69.0%)	60 (30%)	67 (33.5%)
Assumed Response to Question	447	214	230	444	297	275
Test Result Assumption	281	298	231	344	223	250

Total hallucination counts for each agent system on the AgentClinic–MedQA and AgentClinic–MIMIC datasets. “Blocked” refers to hallucinations successfully removed by the Llama-4 post-processing module, whereas “Impact on Diagnosis” refers to hallucinations that were not blocked and ultimately influenced the final diagnostic outcome. Two hallucination types are reported: “Assumed Response to Question” and “Test Result Assumption.” Percentages in

brackets represent the proportion of scenarios in each category (number of scenarios in the category / total scenarios ×100%)
